# Decreased startle modulation during anticipation in the postpartum period in comparison to late pregnancy

**DOI:** 10.1007/s00737-012-0261-7

**Published:** 2012-02-07

**Authors:** Charlotte Hellgren, Elin Bannbers, Helena Åkerud, Victoria Risbrough, Inger Sundström Poromaa

**Affiliations:** 1 Department of Women’s and Children’s Health, Uppsala University, 751 85 Uppsala, Sweden; 2 Department of Psychiatry, University of California, San Diego, CA USA

**Keywords:** Acoustic startle response, Affective modulation, Anticipation, Postpartum, Pregnancy

## Abstract

Knowledge about healthy women’s psychophysiological adaptations during the large neuroendocrine changes of pregnancy and childbirth is essential in order to understand why these events have the potential to disrupt mental health in vulnerable individuals. This study aimed to compare startle response modulation, an objective psychophysiological measure demonstrated to be influenced by anxiety and depression, longitudinally across late pregnancy and the postpartum period. The acoustic startle response modulation was assessed during anticipation of affective images and during image viewing in 31 healthy women during gestational weeks 36–39 and again at 4 to 6 weeks postpartum. No startle modulation by affective images was observed at either time point. Significant modulation during anticipation stimuli was found at pregnancy assessment but was reduced in the postpartum period. The women rated the unpleasant images more negative and more arousing and the pleasant images more positive at the postpartum assessment. Self-reported anxiety and depressive symptoms did not change between assessments. The observed postpartum decrease in modulation of startle by anticipation suggests a relatively deactivated defense system in the postpartum period.

## Introduction

Following childbirth, healthy women go through a number of neuroendocrine alterations which would be considered pathological in non-pregnant, non-postpartal women. These postpartum changes in healthy women include suppression of the hypothalamic–pituitary–adrenal (HPA) axis (Meinlschmidt et al. 2010), attenuated serotonergic activity (Bailara et al. 2006; Doornbos et al. 2008), increased monoamine oxidase A activity (Sacher et al. 2010), and decreased cortical γ-butyric acid (GABA) concentrations (Epperson et al. 2006). However, except from a few studies investigating HPA axis reactivity in relation to social and physical stress (Bessinger et al. 2002; Meinlschmidt et al. 2010), no longitudinal studies have assessed objective psychophysiological measures which may be associated with psychological vulnerability across pregnancy and the postpartum period.

The acoustic startle reflex is a defensive reflex response to a sudden aversive stimulus and is widely used in psychopharmacological animal research in models of fear/anxiety (Davis et al. 1993). Anxiety, fear, and negative affect (threat of shock or aversive images) all potentiate the startle magnitude while it is attenuated by positive affect (reward or pleasant images) (Vrana et al. 1988; Grillon and Baas 2003). Startle potentiation has consistently been shown to involve the amygdala and is also influenced by a number of anxiolytic drugs (Davis et al. 2008). In humans, the affective modulation of the startle response is elevated in experimental models of anxiety (Grillon and Baas 2003; Vrana et al. 1988) and an altered startle response modulation has been demonstrated in anxiety disorders characterized by physical hyperarousal (Grillon et al. 1994; Grillon and Baas 2003; Ray et al. 2009; Lang and McTeague 2009). Depressed patients on the other hand, respond with lower startle modulation by affective images (Dichter and Tomarken 2008; Mneimne et al. 2008).

The startle reflex is also potentiated during the *anticipation* of highly emotional images (Sabatinelli et al. 2001; Dichter et al. 2002). Brain imaging studies on the effect of anticipation highlight activations in the limbic system, primarily the amygdala and the insular cortex (Simmons et al. 2006; Strigo et al. 2008) and depressed patients display less modulation by anticipation stimuli than healthy controls (Dichter and Tomarken 2008; Mneimne et al. 2008).

Data on changes in startle response in response to emotional state and its modulation in human pregnancy and puerperium are sparse. In a previous cross-sectional study, we found no difference in baseline startle response between pregnant women and postpartum women, while pre-pulse inhibition of the startle response was attenuated in the pregnant women (Kask et al. 2008). In non-pregnant women, we have previously shown that the startle response modulation by affect and anticipation is stable across repeated test sessions (Bannbers et al. 2011), why the method was considered suitable for longitudinal use in pregnant/postpartum women.

The aim of this longitudinal study was to compare startle modulation, during emotional image anticipation as well as during viewing of emotional images, in healthy women during late pregnancy and the postpartum period. Based on a number of studies suggesting that the normal postpartum state is accompanied by neuroendocrine and neurotransmitter changes normally seen in depression, we hypothesized that healthy women would display decreased startle modulation by anticipation as well as by emotional images in the postpartum period. Also, since the transition from pregnancy to the postpartum period infers great changes in estradiol and progesterone serum concentrations, a secondary aim was to investigate whether ovarian hormone levels are correlated to emotional and anticipatory startle modulation in this population.

## Materials and methods

### Subjects

Thirty-six healthy pregnant women between the ages of 24 and 39 years were recruited via public maternity health care units in Uppsala County and through local newspaper advertisement. The inclusion criteria were gestational length more than 35 weeks, normal singleton pregnancy, and a planned vaginal delivery. Exclusion criteria were serious pregnancy-related complications (pre-eclampsia, intrauterine growth restriction, gestational diabetes), treatment with psychotropic drugs (including serotonin reuptake inhibitors), and ongoing anxiety and/or depressive disorders during pregnancy. Ongoing psychiatric disorders were evaluated using the Swedish version of the Mini International Neuropsychiatric Interview, a structured interview based on DSM-IV and ICD-10 (Sheehan et al. 1998). In addition, exclusion criteria for the postpartum visit were severe delivery or postpartum complications (mother and/or fetus), and more than 50% non-responses at the first test session. Four women were non-responders, and one woman was excluded due to an obstetric complication. Hence, at the postpartum test session, 31 women with valid startle data and no severe obstetric complications remained eligible and consented to a second visit. The excluded women did not differ from the remaining study population in terms of age, parity, BMI, MADRS-S, and STAI-S scores (excluded 30.8 ± 3.4 vs. included 30.1 ± 3.8 years, 0.4 ± 0.9 vs. 0.4 ± 0.7 prior deliveries, 24.9 ± 4.7 vs. 23.9 ± 4.2 kg/m^2^; MADRS-S, 6.2 ± 3.2 vs. 6.3 ± 4.8; STAI-S, 32.4 ± 3.4 vs. 29.1 ± 6.4).

The study procedures were in accordance with ethical standards for human experimentation, and the study was approved by the Regional Ethical Review Board, Uppsala University, Sweden. Written informed consent was obtained from all subjects before inclusion.

### Study procedure

All tests were carried out at the Department of Women’s and Children’s Health, Uppsala University between 0800 and 1800 hours. The first test session was scheduled approximately 14 days before the predicted day of delivery, based on routine ultrasound examination in gestational weeks 16–17. The second test session took place 4 to 6 weeks postpartum and was scheduled based on the date of delivery. All visits were made between January and August 2009. One researcher conducted all test sessions.

At the pregnancy test session, subjects were interviewed about obstetric history, medication during the preceding 3 months, and tobacco and alcohol use. At both visits, all subjects filled out the Montgomery-Åsberg Depression Rating Scale–Self-rated version, MADRS-S (Svanborg and Asberg 1994) and the Spielberger State–Trait Anxiety Inventory–State version, STAI-S (Spielberger 1983). Subjects also filled out the Edinburgh Postnatal Depression Scale, EPDS (Cox et al. 1987) at the postpartum test session.

### Acoustic startle response assessment

The acoustic startle response was assessed by electromyographic (EMG) recording of the right musculus orbicularis oculi. The acoustic startle probes and the recording of the eye blink responses were controlled by a commercial startle system (SR-HLAB, San Diego Instruments, San Diego, CA, USA). Acoustic startle probes were delivered binaurally by headphones (TDH-39-P, Maico, Minneapolis, MN, USA). The sound was calibrated with a Quest Electronics meter (model 210 Quest Technologies, Oconomov, WI, USA). After the skin was scarified with alcohol, two miniature silver/silver chloride electrodes (In Vivo Metric, Healdsburg, CA, USA) with a small amount of electrode gel (Sigma gel, In Vivo Metric, CA, USA), were positioned below the subject’s right eye, over the orbicularis oculi muscle. A ground electrode was placed in the center of the forehead (Blumenthal et al. 2005). Electrode impedances were confirmed to be less than 5 kOhm. The EMG was filtered (100–1,000 Hz), digitized at 1 kHz and analyzed by the EMG startle response software which rectified and smoothed the EMG response with a 10-ms time constant.

The subjects sat upright in an armchair and were instructed to watch a 14.1-inch computer monitor positioned approximately 1 m in front of the subject. The participants were aware that they were going to see pleasant and unpleasant pictures and hear unpleasant but harmless sound pulses throughout the experiment. The lights were switched off and the researcher left the room during testing. The test session began with a 5-min acclimation period without startle probes or images. Following the acclimation period, a 10-min slide show was displayed on the monitor while semi-randomized startle probes, 105-dB 40-ms broadband white noise with instantaneous rise time, were delivered. The startle modulation paradigm in this study is analogous to an image anticipation task previously used in a number of imaging studies of neural circuit activation across pleasant and unpleasant anticipation (Simmons et al. 2009; Aupperle et al. 2011). Each session consisted of 34 blocks containing three different startle conditions: (1) a black screen during which baseline startle response was measured (control condition), (2) a red or green screen as the negative or positive anticipation stimulus, (3) an unpleasant or pleasant image. A red screen always preceded unpleasant images and a green screen always preceded pleasant images. Across the 34 blocks, a total number of 48 startle probes were delivered; 20 during the control condition, seven during positive anticipation stimuli, seven during negative anticipation stimuli, seven during pleasant image stimuli, and seven during unpleasant image stimuli. The pleasant and unpleasant image blocks were counterbalanced. The control condition was presented for variable times, ranging between 8 and 13 s, while the anticipation stimuli were presented for 6 s and the image stimuli were presented for 2 s. Startle probes were delivered 4 to 10 s after the onset of the black screen (control condition), 1.5 to 4.5 s after the onset of the anticipation stimuli, and 0.5 to 1.5 s after onset of the image stimuli. To enable exclusion of trials with excessive background noise, 22 amplitude recordings were made when no startle probe was delivered (non-stimulus recordings).

The images were obtained from the International Affective Picture System (IAPS) (Lang et al. 2005). The pictures were selected to be pleasant or unpleasant, with 34 pictures from each category (specific numbers are listed in Table 1) divided in two series (A and B) with equal normative ratings of valence and degree of arousal according to the IAPS manual (Lang et al. 2005). Half of the study group was presented with image series A during pregnancy and series B on their postpartum assessment, while the other half saw the image series in opposite order. Previous studies have shown that no habituation in startle modulation, either by images or by anticipation, occurs when the affective images are changed between sessions (Larson et al. 2000; Bannbers et al. 2011). After every session, the subjects were asked to rate the image valence and arousal. Valence was rated on 10-cm visual analogue scales ranging from positive (0.0 cm) to negative (10.0 cm). Arousal ratings ranged between calm (0.0 cm) and aroused (10.0 cm).

**Table 1:** Numbers of the International Affective Picture System (IAPS) pictures (Lang et al. 2005)

Type	Series	Picture number
Pleasant	A	1440, 1463, 1710, 1811, 1920, 2057, 2070, 2152, 2165, 4599, 4641, 5629, 5831, 7330, 8190, 8200, 8370
	B	1460, 1540, 1721, 1722, 2071, 2160, 2260, 2332, 2341, 2398, 2655, 2660, 5260, 5621, 5833, 5910, 8499
Unpleasant	A	1026, 1220, 1301, 1932, 2205, 3030, 3051, 3060, 3071, 3160, 3181, 3220, 3350, 9040, 9405, 9410, 9433
	B	1050, 1052, 1111, 1201, 1300, 1525, 1930, 1931, 3000, 3015, 3102, 3170, 3261, 6415, 9300, 9570, 9571

### Steroid hormone analyses

Venous blood samples were collected after the startle sessions. The samples were centrifuged at 1,500 × *g* for 10 min and stored at −20°C within an hour after sampling. The steroid hormone analyses were carried out by competitive immunometry electrochemistry luminescence detection at the Department of Clinical Chemistry, Uppsala University hospital. The samples were run on a Roche Cobas e601 with Cobas Elecsys estradiol and progesterone reagent kits, respectively (Roche Diagnostics, Bromma, Sweden). For progesterone, the measurement interval was 0.1–191 nM and for estradiol 18.4–15,781 pM. Thus, the serum samples taken in late pregnancy were diluted 1:100. Progesterone intra-assay coefficient of variation was 2.2% at 2.39 nmol/L and 2.8% at 31.56 nmol/L. The total coefficient of variation was 4.8% at 2.52 nmol/L and 2.0% at 112 nmol/L. Estradiol intra-assay coefficient of variation was 6.8% at 85.5 pmol/L and 2.8% at 1,640 pmol/L. The total coefficient of variation was 4.7% at 120 pmol/L and 2.6% at 12,935 pmol/L.

### Statistical analyses

Peak startle amplitudes were measured automatically within 20–150 ms following the onset of the startle probe. A zero response score was given if no response was detected according to the default criteria provided by the software: (1) the peak startle response occurred outside the 20–150-ms time frame (2) a baseline shift exceeded 40 arbitrary units, or (3) a startle response was 25 arbitrary amplitude units or less. Of the responses, 3.0% were scored as zero responses, and they were evenly distributed between the pregnant and postpartum states. Startle magnitude was defined as the total amplitude of all trials with response/total number of trials.

Because mean baseline startle response differed by a factor of 10 between individuals, z-scores standardized to the control condition within each subject and test session for pleasant and unpleasant images, and positive and negative anticipation were used in the analyses (magnitude of each startle − mean magnitude of control startles)/standard deviation of magnitude control startles. The startle magnitude during the four different stimuli was compared between the pregnancy and postpartum states by use of two-way ANOVA with repeated measures. Within subjects factors in the ANOVA analyses were state (pregnancy vs. postpartum period) and stimulus (positive anticipation stimuli vs. negative anticipation stimuli or pleasant images vs. unpleasant images). Differences between pregnant and postpartum states in baseline startle response (control condition), MADRS-S scores and STAI-S were compared with Mann–Whitney *U* tests and chi-square tests. All values in the text are displayed as mean ± SD, unless otherwise stated. The SPSS statistical package was used for the analyses. *P* values of less than 0.05 were considered to be statistically significant.

## Results

The 31 included women were 30.1 ± 3.8 years old and 21 (67.7%) were nulliparous. The test sessions took place 15.9 ± 7.5 days before delivery and 35.6 ± 5.3 days following parturition. No women reported use of alcohol or tobacco during pregnancy, and when examined during the postpartum period, all but one were breast feeding. The STAI-S and MADRS-S did not differ between the pregnant and postpartum states (STAI-S: pregnant, 29.1 ± 6.4 vs. postpartum, 28.7 ± 6.9; MADRS-S: pregnant, 4.8 ± 0.8 vs. postpartum, 4.3 ± 0.8). The mean postpartum EPDS score was 4.5 ± 4.2.

### Modulation of acoustic startle magnitude

Startle magnitude during the control condition did not differ between the pregnant and postpartum state, Table 2 *.* A significant main effect of stimulus was found (*F*(3,28) = 9.50; *p* < 0.001). Post hoc test revealed significant decreases in startle response between the control condition and the pleasant image stimuli (*F*(1,30) = 7.40; *p* < 0.05) and the positive anticipation stimuli (*F*(1,30) = 26.05; *p* < 0.001), whereas there was no difference between the control condition and the negative image stimuli (*F*(1,30) = 1.72; *p* = 0.20) and negative anticipation stimuli (*F*(1,30) = 2.41; *p* = 0.13), respectively, Table 2. Startle magnitude differed between the positive and negative anticipation stimuli (*F*(1,30) = 25.19; *p* < 0.001), but not between the pleasant and unpleasant image stimuli (*F*(1,30) = 3.98; *p* = 0.055). No difference in startle modulation was observed between image series A and B and the non-stimulus recordings revealed no significant background noise.

**Table 2:** Mean ± SD startle magnitudes during the five different conditions at the pregnant and postpartum assessments

	Control condition	Positive images	Negative images	Positive anticipation	Negative anticipation
Pregnant, AU	1888 ± 1239	1672 ± 1153	1712 ± 1285	1632 ± 1246	2058 ± 1336
Postpartum, AU	1844 ± 1259	1721 ± 1197	1823 ± 1239	1737 ± 1263	1880 ± 1256

*AU* arbitrary units

### Difference in startle modulation between the pregnant and postpartum state

**Fig. 1:**
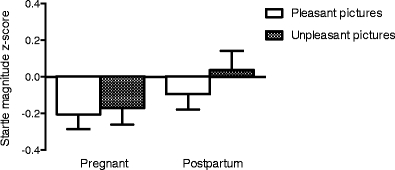
Mean z-scores ± S.E.M. of the acoustic startle response during pleasant and unpleasant images in 31 women examined during late pregnancy and in postpartal week 4 to 6. No difference in startle magnitude response to pleasant and unpleasant image stimuli across the pregnant and postpartum states was evident (main effect of state *F*(1,30) = 1.99; *p* = 0.169, state by stimulus interaction *F*(1,30) = 0.48; *p* = 0.492)

No difference in startle magnitude response to pleasant and unpleasant image stimuli across the pregnant and postpartum states was evident (main effect of state *F*(1,30) = 1.99; *p* = 0.169, state by stimulus interaction *F*(1,30) = 0.48; *p* = 0.492), Fig. 1.

**Fig. 2:**
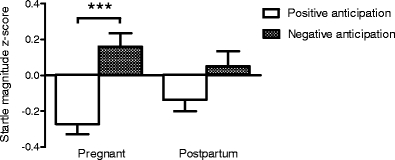
Mean z-scores ± S.E.M. of the acoustic startle response during anticipation of pleasant and unpleasant images in 31 women examined during late pregnancy and in postpartal week 4 to 6. The ANOVA showed that the state by stimulus effect was significant (*F*(1,30) = 4.86; *p* = 0.035). There was a significant difference between the positive and negative anticipation conditions during late pregnancy (*F*(1,30) = 36.91; *p* < 0.001) but not at the postpartum assessment (*F*(1,30) = 4.06)

As depicted in Fig. 2, a significant pregnancy/postpartum state by stimulus interaction for the positive and negative anticipation stimuli was found (*F*(1,30) = 4.86; *p* = 0.035), illustrated by a significant difference between the positive and negative anticipation conditions during late pregnancy (*F*(1,30) = 36.91; *p* < 0.001) but not at the postpartum assessment (*F*(1,30) = 4.06), Fig. 2.

### Correlations with steroid hormones

As expected, the serum concentrations of estradiol and progesterone declined between the pregnancy and postpartum states (estradiol: pregnant, 108,063 ± 36,057 pmol/L; postpartum, 76 ± 56 pmol/L; progesterone: pregnant, 692.8 ± 240.6 nmol/L; postpartum, 1.0 ± 0.5 nmol/L). The difference between positive and negative anticipation stimuli (Δ–anticipation startle response) was significantly and positively correlated with progesterone levels in the postpartum state (*r* = 0.405, *p* = 0.024) but there was no correlation with estradiol levels (*r* = 0.01, *p* = 0.97). No correlations between progesterone- or estradiol serum concentrations and Δ–anticipation startle response were found during the pregnant state (progesterone: *r* = 0.09, *p* = 0.61; estradiol: *r* = 0.17, *p* = 0.35).

### Ratings of IAPS images

Mean valence and arousal scores are displayed in Table 3. A significant interaction was found between pregnancy/postpartum states and valence ratings: *F*(1,29) = 10.11; *p* < 0.01. The women rated the unpleasant images more negative and the pleasant images more positive at the postpartum visit than during pregnancy (*p* < 0.05, respectively). There was also a significant interaction between pregnant/postpartum states and arousal ratings: *F*(1,29) = 12.91; *p* = 0.001. The women indicated increased arousal scores for unpleasant images at their postpartum visit (*p* < 0.01), whereas there was no difference in arousal scores between pregnancy and the postpartum period for the pleasant images.

**Table 3:** The study subjects’ visual analogue valence and arousal ratings of the IAPS images

	Late pregnancy	Postpartum
Valence, pleasant images	1.9 ± 0.9	1.6 ± 0.7*
Valence, unpleasant images	7.8 ± 1.1	8.1 ± 1.0*
Arousal, pleasant images	2.9 ± 1.8	2.6 ± 1.8
Arousal, unpleasant images	5.7 ± 1.9	6.4 ± 1.6**

Data is presented as mean ± S.D. Valence was rated on 10-cm visual analogue scales ranging from positive (0.0 cm) to negative (10.0 cm). Arousal ratings were made from calm (0.0 cm) to aroused (10.0 cm)

*p < 0.05; **p < 0.01, compared with late pregnancy

## Discussion

Modulation of startle by negative and positive image anticipation decreased during the puerperium in comparison with late pregnancy. This decrease was not accompanied by any changes in self-reported anxiety or mood symptoms, but the postpartum anticipatory startle modulation was correlated with serum progesterone levels. We also confirm our prior finding of unchanged baseline startle response in women examined during late pregnancy and the postpartum period. However, no startle modulation was detected during affective image viewing in late pregnancy or in the puerperium.

The major change observed in the present study across late pregnancy and the postpartum state was decreased startle modulation during anticipation in the postpartum period. The anticipatory startle modulation range in pregnant women was similar to what has previously been reported in healthy women across the menstrual cycle (Bannbers et al. 2011). Hence, we interpret the startle modulation during anticipation in pregnant women as normal, whereas the low postpartum startle modulation is interpreted as deviant. Moreover, the decrease in startle modulation during anticipation occurred even though the women, at this time point, rated the negative images significantly more negative and more arousing. As increased negative arousal normally potentiates the startle response (Cuthbert et al. 1996), it could have been anticipated that postpartum women would display an increased anticipatory startle modulation. Automatic physiological responses (as measured by startle) to affective stimuli have been reported to be low in high anxiety populations, despite higher subjective ratings of arousal of these stimuli. This discrepancy is suggested to be due to an exhausted defense system (Lang and McTeague 2009). Possibly, the neuroendocrine changes of the postpartum period result in a similarly deactivated defense system, evidenced by an attenuated startle response modulation. This observation is in line with the attenuated stress response seen in breast-feeding women (Altemus et al. 2001). However, the stress reactivity of both the HPA axis and the sympathetic nervous system are blunted also during late pregnancy (Kammerer et al. 2002; DiPietro et al. 2005; Hellgren et al. 2011), which underlines the need for more longitudinal studies to assess the extent and characteristics of defense system attenuation across pregnancy and the postpartum period.

Furthermore, the anticipatory startle modulation range in postpartum women was positively correlated with progesterone levels. As it may be inferred that progesterone production in lactating postpartum women is predominantly of adrenal origin (Wirth et al. 2007), this finding may suggest that modulation of startle during affective anticipation is dependent on HPA axis reactivity, although cortisol has previously been associated with elevations only in baseline startle (Buchanan et al. 2001). Brain imaging studies on the effect of anticipation highlight activations in the limbic system, primarily in the amygdala and insula. For example, the anticipation of pain evokes less activity in the insula of depressed individuals than in healthy controls (Strigo et al. 2008). Anxiety-prone subjects on the other hand, have a higher insular activation during image anticipation relative to control subjects (Simmons et al. 2006). The limbic system is rich in estrogen receptors (Östlund et al. 2003) and progesterone (Bixo et al. 1997) in women, and it is possible that the receptor expression and responsivity is altered during periods of low progesterone levels*.* Other pathways which influence the startle response include the GABA and serotonin neurotransmitter systems (Harmer et al. 2006; Baas et al. 2009), which are both altered in the postpartum period (Doornbos et al. 2008; Bailara et al. 2006; Epperson et al. 2006). Decreased startle modulation may thus represent an adaptive change in emotional reactivity at a time of rapid and dynamic neuroendocrine changes.

The women in this study displayed attenuated startle in response to stimuli signaling the anticipation of pleasant pictures, which is partly at odds with previous work on startle during picture anticipation (Dichter et al. 2002; Sabatinelli et al. 2001), although see (Nitschke et al. 2002). This result likely reflects the difference in arousal between our picture categories. The pictures were chosen to have similar normative arousal ratings across categories, but we refrained from using explicit sexual content and thus used pictures with lower arousal ratings for the pleasant pictures than for the unpleasant. The pleasant pictures can therefore be considered close to neutral and the modulation during negative anticipation is hence in line with existing literature.

The lack of significant startle modulation by pleasant and unpleasant images during pregnancy and the postpartum period is surprising in relation to existing literature (Vrana et al. 1988; Grillon and Baas 2003) and our previous experience. A contributing factor could be that the images were shown for a shorter time than the anticipation stimuli as the paradigm used in the study is primarily designed to assess anticipation. By use of the same experimental setup, we have previously observed a significant startle modulation also during pleasant compared to negative images in healthy women and in women with premenstrual dysphoric disorder (Bannbers et al. 2011). One explanation to the lower modulation during pregnancy and postpartum could be that the image onset may act as a weak pre-pulse that decreases startle magnitude (Bradley et al. 1993). Previous data from our group, however, suggest that pregnant women have lower levels of pre-pulse inhibition than postpartum women (Kask et al. 2008). Possibly, pregnant as well as postpartum women are more sensitive to pre-pulse inhibition by images than the non-pregnant controls. Another explanation is that both late pregnancy and the postpartum period are associated with attenuated modulation by affective images. These explanations must be tested in future studies using a non-pregnant control group.

Research on psychophysiological responses across pregnancy and the postpartum period is rare, and we believe that the current longitudinal study is the first on startle modulation by affect and anticipation in this population. In conclusion, we found decreased startle modulation by anticipation in the puerperium in comparison with late pregnancy, possibly indicating altered responsiveness of the limbic system and thereby the autonomous defense system. Further studies evaluating startle and its modulation in women who do develop anxiety and/or depressive disorders during pregnancy and the postpartum period are necessary to fully understand the significance of this finding.
